# P-317. Efficacy of CCR5 Gene Editing via CRISPR-Cas9 in HIV/AIDS Prevention: A Meta-Analysis

**DOI:** 10.1093/ofid/ofaf695.536

**Published:** 2026-01-11

**Authors:** Matheus Santos Samaritano Pereira

**Affiliations:** Universidade Municipal de São Caetano do Sul, Boituva, Sao Paulo, Brazil

## Abstract

**Background:**

HIV-1 remains a major global health challenge. The CCR5 receptor on CD4+ T cells is key for viral entry. Individuals homozygous for the CCR5Δ32 mutation show natural resistance to HIV, suggesting that gene inactivation may offer protection. CRISPR-Cas9 editing has emerged as a strategy to mimic this effect. This meta-analysis evaluates the efficacy and safety of CCR5 editing via CRISPR-Cas9 in HIV-1 prevention.

**Figure:**
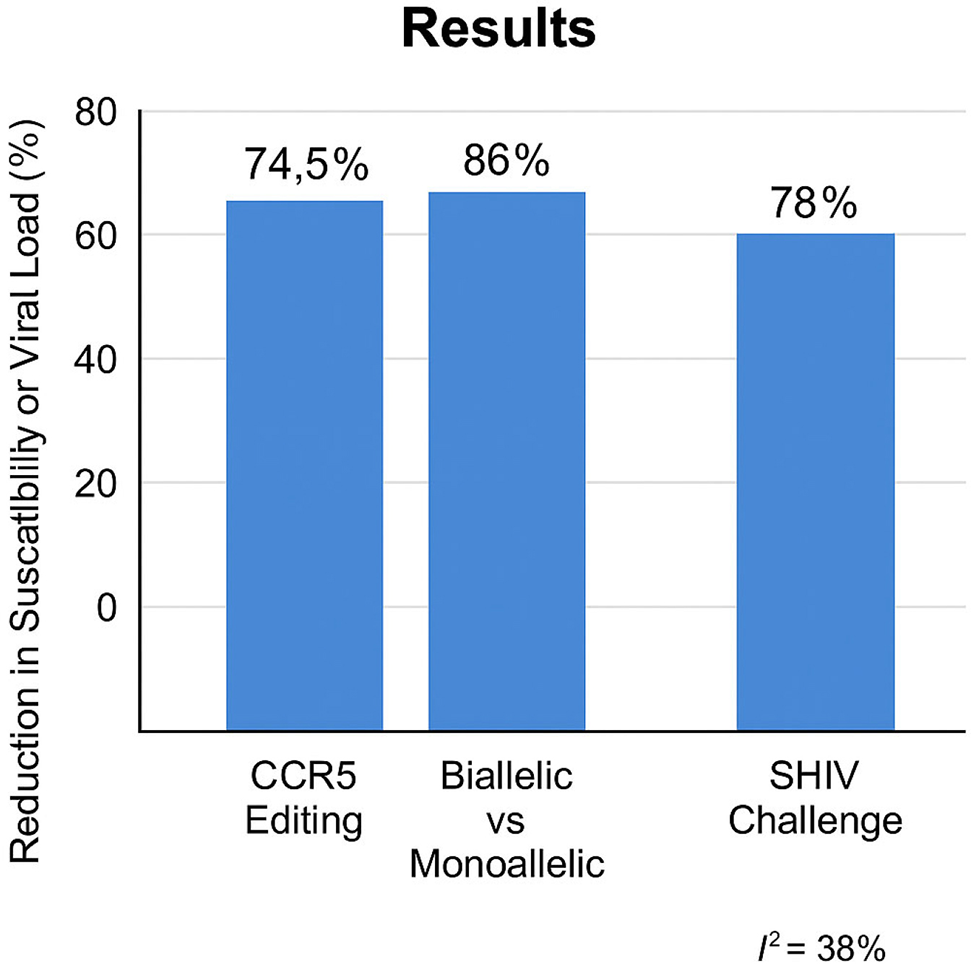
Summary of Meta-Analysis Results Bar graph showing key findings from the meta-analysis: 74.5% reduction in HIV-1 susceptibility after CCR5 editing, 86% higher protection in biallelic versus monoallelic editing, and 78% viral load decrease following SHIV challenge.

**Figure:**
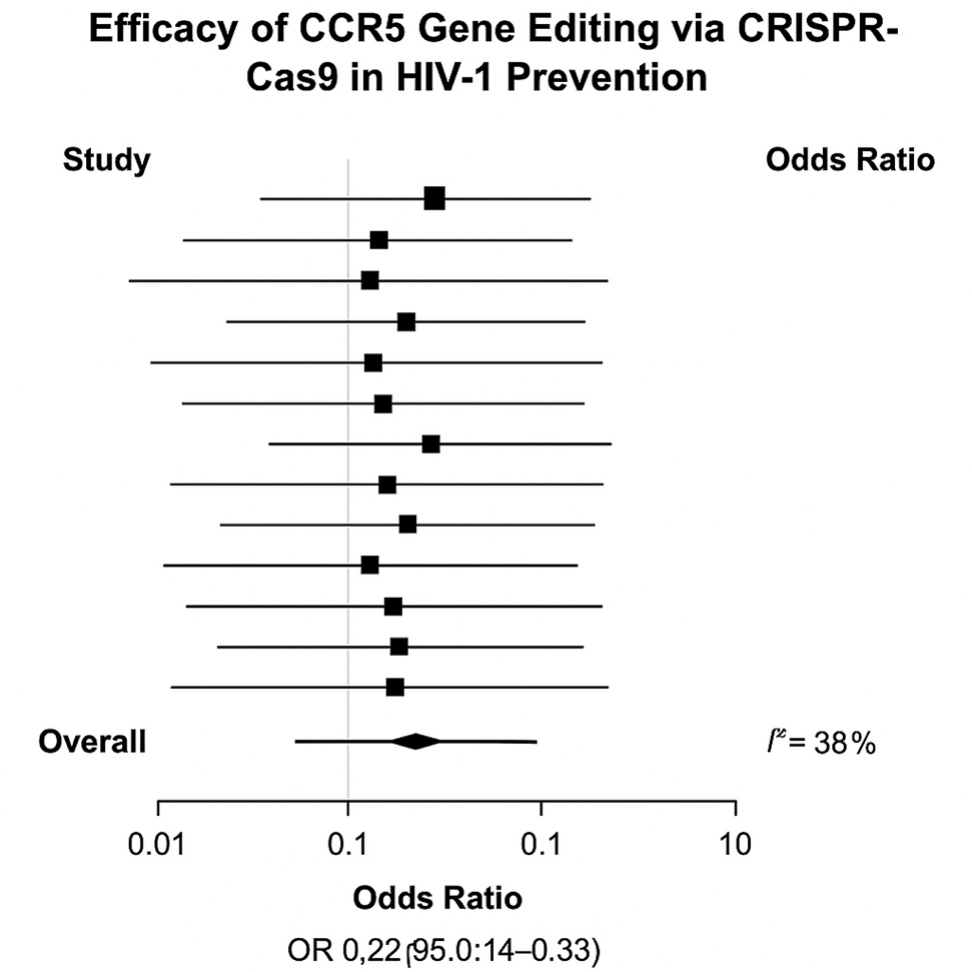
Forest Plot of CCR5 Gene Editing Effectiveness

Forest plot illustrating the pooled odds ratio (OR=0.22; 95% CI: 0.14–0.33; p<0.0001) for HIV-1 susceptibility reduction following CRISPR-Cas9-mediated CCR5 editing. Moderate heterogeneity was observed (I²=38%).

**Methods:**

A systematic review was conducted following PRISMA 2020 guidelines. A comprehensive search of PubMed, Embase, Web of Science, and Scopus was performed, including studies published until March 2025. In vivo and in vitro studies, including animal models and human trials, that used CRISPR-Cas9 for knockout or functional disruption of CCR5 were included. The primary outcome was the reduction in susceptibility to HIV-1 infection. Study quality was assessed using the SYRCLE and RoB 2.0 tools. A random-effects model was used for meta-analysis, with calculation of odds ratios (OR), relative risk reduction (RRR), and heterogeneity (I²). Sensitivity and subgroup analyses were performed.

**Results:**

Twelve studies met inclusion criteria (n=731 samples). CCR5 editing resulted in a 74.5% reduction in HIV-1 susceptibility (RRR=74.5%; OR=0.22; 95% CI: 0.14–0.33; p< 0.0001). The heterogeneity between studies was moderate (I²=38%). Subgroup analysis revealed that biallelic CCR5 editing conferred superior protection, with an OR of 0.14 (95% CI: 0.08–0.25) compared to monoallelic editing (OR=0.35; 95% CI: 0.22–0.55). Additionally, models employing CCR5 editing followed by SHIV challenge demonstrated a 78.3% decrease in viral load in comparison to non-edited controls (p< 0.0001). Long-term follow-up (≥6 months) in animal models showed sustained protection without detectable off-target effects or genomic instability. No severe adverse events related to gene editing were reported across studies.

**Conclusion:**

CRISPR-Cas9-mediated CCR5 editing demonstrated substantial efficacy in preventing HIV-1 infection, with a remarkable 74.5% reduction in susceptibility and no significant safety concerns. Biallelic editing was more effective than monoallelic, and sustained long-term protection without adverse effects was observed.

**Disclosures:**

All Authors: No reported disclosures

